# Effect of Ethanol on Brain Electrical Tissue Conductivity in Social Drinkers

**DOI:** 10.1002/jmri.29548

**Published:** 2024-08-06

**Authors:** Jun Cao, Iain K. Ball, Elizabeth Summerell, Peter Humburg, Tom Denson, Caroline D. Rae

**Affiliations:** ^1^ Neuroscience Research Australia Sydney New South Wales Australia; ^2^ Philips Australia & New Zealand North Ryde New South Wales Australia; ^3^ School of Psychology, The University of New South Wales Sydney New South Wales Australia; ^4^ Mark Wainwright Analytical Centre, Stats Central, The University of New South Wales Sydney New South Wales Australia

**Keywords:** electrical properties tomography, brain activity, alcohol

## Abstract

**Background:**

How the biophysics of electrical conductivity measures relate to brain activity is poorly understood. The sedative, ethanol, reduces metabolic activity but its impact on brain electrical conductivity is unknown.

**Purpose:**

To investigate whether ethanol reduces brain electrical tissue conductivity.

**Study Type:**

Prospective.

**Subjects:**

Fifty‐two healthy volunteers (aged 18–37 years, 22 females, 30 males).

**Field Strength/Sequence:**

3 T, T1‐weighted, multi‐shot, turbo‐field echo (TFE); 3D balanced fast‐field echo (bFFE).

**Assessment:**

Brain gray and white matter tissue conductivity measured with phase‐based magnetic resonance electrical properties tomography (MREPT) compared before and 20 minutes after ethanol consumption (0.7 g/kg body weight). Differential conductivity whole brain maps were generated for three subgroups: those with strong ($∆\sigma_{\max}$ > 0.1 S/m; N = 33), weak (0.02 S/m ≤ $∆\sigma_{\max}$ ≤ 0.1 S/m; N = 9) conductivity decrease, and no significant response ($∆\sigma_{\max}$ < 0.02 S/m, N = 10). Maps were compared in the strong response group where breath alcohol rose between scans, vs. those where it fell.

**Statistical Tests:**

Average breath alcohol levels were compared to the differential conductivity maps using linear regression. T‐maps were generated (threshold *P* < 0.05 and *P* < 0.001; minimum cluster 48 mm^3^). Differential conductivity maps were compared with ANOVA.

**Results:**

Whole‐group analysis showed decreased conductivity that did not survive statistical thresholding. Strong responders (N = 33) showed a consistent pattern of significantly decreased conductivity ($∆\sigma_{\max}$ > 0.1 S/m) in frontal/occipital and cerebellar white matter. The weak response group (N = 9) showed a similar pattern of conductivity decrease (0.02 S/m ≤ $∆\sigma_{\max}$ ≤ 0.1 S/m). There was no significant relationship with breath alcohol levels, alcohol use, age, ethnicity, or sex. The strong responders' regional response was different between ascending (N = 12) or descending (N = 20) alcohol during the scan.

**Data Conclusion:**

Ethanol reduces brain tissue conductivity in a participant‐dependent and spatially dependent fashion.

**Evidence Level:**

1

**Technical Efficacy:**

Stage 2

As one of the most widely used drugs in the Western world, ethanol is a known sedative and depressant with a broad range of actions in the brain.^1^ Acute consumption of ethanol induces a range of physiological, psychological, and behavioral effects,^2^ some of which are dose‐dependent and some of which display individual differences in their effect.^3^ As a two‐carbon, small molecule, the interaction of ethanol with other molecules is relatively non‐specific, occurring through hydrogen bonding and weak hydrophobic interactions.^4^ Consequently, the number of reported pharmacological interactions of ethanol with the nervous system is broad, with reported interactions at multiple neurotransmitter receptors,^4^ as well as an array of less specific interactions including interactions with brain lipids, although these are likely to occur at very high (>>100 mM) concentrations of ethanol,^5^ which are typically outside of the usual consumption ranges.

The effects of ethanol administration are known to be variable, producing both stimulant and sedative responses with the stimulant responses generally occurring early in the time course of consumption and at low concentrations, while the sedative effects occur later and in response to higher levels of consumption.^6^

Acute administration of ethanol is known to reduce brain metabolism (glucose uptake) 40 minutes after consumption with a ceiling effect (beyond which glucose uptake does not further decrease) at around 1 g/kg body weight alcohol.^7^ The effects of ethanol following consumption take time (around 40 minutes) to have full effect with low dose (0.25 g/kg), producing decreased uptake of Fluorodeoxyglucose (FDG) in some subjects but not others.^7^

Advances in the radiofrequency stability of MRI scanners has meant that phase‐based MR electrical properties tomography (MREPT) now has sufficient precision to be of use as a quantitative research approach for the measurement of brain electrical properties, such as tissue electrical conductivity.^8^ Electrical conductivity, which reflects a material's ability to conduct electric field, is one of the fundamental properties of materials, and is particularly relevant in an electrical tissue such as the brain. It has been observed to increase in response to brain activity,^9^ with previous work suggesting that the baseline (resting) value of electrical conductivity may be related to the level of (resting) brain activity.

Against this background, we aimed to assess whether tissue electrical conductivity changes would reflect decreases in brain activity following alcohol consumption.

## Materials and Methods

This study was approved by the institutional Human Research Ethics Committee (HC200014). Written informed consent was obtained from all participants.

### Participants

This prospective study included 52 healthy young participants (age range: 18–37 years, median 19 years; 22 females, 30 males), who were assessed as social drinkers if they reported consuming alcohol at least once per month and their scores of the Alcohol Use Disorders Identification Test (AUDIT)^10^ were not larger than 15. Participants belonged to the following ethnicities: Asian (69.23%), Caucasian (19.23%), other (11.54%). All participants self‐identified as right‐handed. Participants were asked to eat something prior to attending the scanning session to avoid possible nausea.

### Protocol

**FIGURE 1:**
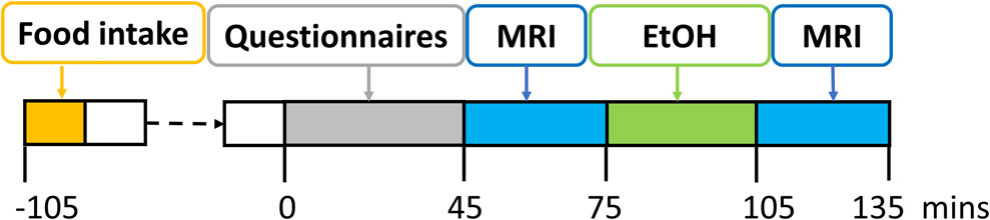
Study timeline. EtOH = ethanol.

MRI scans were acquired between 9.45 am and 2.30 pm (median time at 10.15 am). Following removal from the scanner, participants received two alcoholic beverages containing a combined total of 0.70 grams of ethanol per kg of body weight. Each drink contained a ratio of 1:3 vodka (37% ABV) to sugar‐free lemonade. A maximum dose of 50 g alcohol (five standard drinks) was administered to reduce the possibility of nausea. Breath alcohol readings (g alcohol/210 L breath) were recorded with a calibrated Alcolizer LE5 (Alcolizer Technology, Balcatta, WA, Australia). Twenty minutes after ethanol administration, participants were scanned again. The protocol outline is shown in Fig. 1.

### MRI—Acquisition

All images were acquired at 3 T using a 32‐channel digital receive head coil and parallel transmit (Ingenia CX; Philips Healthcare, Best, The Netherlands). A structural three‐dimensional (3D) T1‐weighted, multi‐shot, turbo field echo (TFE) image (repetition time [TR]/echo time [TE] = 7.10/3.38 msec, resolution 1 × 1 × 1 mm^3^, sagittal slices, field of view [FOV] 240 × 240 × 170 mm^3^, flip angle 8°, nonselective radiofrequency [RF] pulses) was collected for anatomical coregistration and tissue parcellation. RF shimming calibrated with B1+ mapping using full‐coverage, two‐dimensional (2D) dual refocusing echo acquisition mode (DREAM) was then applied.^11^ A balanced, fast‐field echo (bFFE; TR/TE = 2.52/1.26 msec, nonselective RF pulses, flip angle 25°, resolution 1 × 1 × 1 mm^3^, sagittal slices, FOV 240 × 240 × 190 mm^3^) was collected along with the associated phase map for determination of phase‐based tissue conductivity.

### MRI—Analysis

Phased‐based MREPT was adopted as $\sigma \approx \nabla^{2}\varphi_{\pm }/2\mu_{0}\omega$, where σ was conductivity, $\varphi_{\pm }$ denoted the transceive phase, $\mu_{0}$ was the magnetic permeability of free space, ω was angular frequency (Larmor frequency), and $\nabla^{2}$ was the Laplacian operator. T1‐weighted TFE images were co‐registered and segmented into white matter, gray matter, and cerebrospinal fluid (CSF) using FSL (https://fsl.fmrib.ox.ac.uk/fsl/fslwiki) to alleviate boundary artifacts in the calculation. Within each tissue type, an average parabolic phase fitting method was used to reduce artifacts amplified in the Laplacian,^11^, ^12^ and the second derivatives of the fitted phase were taken to calculate conductivity.

The conductivity maps of each participant from both sessions were normalized into Montreal Neurological Institute (MNI) space (voxel size 2 mm isotropic) using SPM 12 (https://www.fil.ion.ucl.ac.uk/spm/), and the differential conductivity map of each participant was obtained by subtracting the normalized conductivity map of the post‐alcohol session from that of the pre‐alcohol session. No spatial smoothing was applied. After statistical analysis (section below), the T maps were overlain on the Desikan‐Killiany atlas, Automatic anatomical labeling (AAL) atlas, and Natbrainlab white matter atlas^13^ using MRIcroGL (v1.2.20220720; https://www.nitrc.org/projects/mricrogl) to verify anatomical regions.

Regarding banding artifact removal, bFFE imaging suffers from banding artifacts, which often occur at locations where the main field inhomogeneity is a multiple of 1/TR. Short‐TR bFFE greatly reduces banding artifacts and preserves the quality of its phase image.^14^ However, banding artifacts were still observed in tissue‐air interfaces (mainly rectus gyrus) from some participants, particularly those with larger head volumes. Banding artifacts were identified automatically by values of phase >2.5 rad as the phase of the banding artifacts areas was close to positive π (3.14) rad while their surrounding tissue had negative phase values. The binary masks of banding artifacts were extracted from all participants, and normalized into MNI space (voxel size 2 mm isotropic) using SPM 12 (https://www.fil.ion.ucl.ac.uk/spm/). The union of the normalized binary masks of banding artifacts from all participants was taken as the ultimate mask of banding artifacts. The banding artifacts region and its adjacent voxels were masked to ensure the results were not biased by this artifact.

### Statistical Analysis

Principal components analysis (PCA) was conducted in SIMCA P+ v 11.5 (Umetrics, Umeå, Sweden). Statistical analysis of conductivity maps was performed in MATLAB R2023b, tissue segmentation was performed in FSL 6.0.3 (https://fsl.fmrib.ox.ac.uk/fsl/fslwiki).

After statistical analysis, the T maps were overlain on the Desikan‐Killiany atlas, AAL atlas, and Natbrainlab white matter atlas using MRIcroGL (v1.2.20220720; https://www.nitrc.org/projects/mricrogl) to verify anatomical regions.

To determine whether variables age, sex, ethnicity or AUDIT score were related to breath alcohol content (BrAC) before and after the scan, these variables were subjected to PCA to identify if there were any relationships between these variables. Similarly, PCA was used to determine whether or not there was any relationship between BrAC, sex, ethnicity and AUDIT score, and membership of response group (strong, weak or non‐responder).

The two measurements of breath alcohol content prior to and post the second MRI scan session were averaged for each participant. The differential conductivity maps and the mean breath alcohol content measurements from 52 participants were analyzed using linear regression in SPM 12 (https://www.fil.ion.ucl.ac.uk/spm/), with age as a covariate, to determine if conductivity change was significantly related to breath alcohol content. Unthresholded T maps and thresholded T maps (*P* < 0.05 and *P* ≤ 0.001) were generated.

The participants were categorized into three groups based on their maximum conductivity change $∆\sigma_{\max}$ after clustering with a minimum volume size of 48 mm^3^ (33 strong responders: $∆\sigma_{\max}$ > 0.1 S/m; 9 weak responders: 0.02 S/m ≤ $∆\sigma_{\max}$ ≤ 0.1 S/m; 10 non‐responders: $∆\sigma_{\max}$ < 0.02 S/m). The value of 0.1 S/m was selected as this is the amount that tissue conductivity has been shown to increase following performance of a task such as visual stimulation or fist clenching,^9^, ^15^ indicating that a corresponding decrease of the same magnitude is likely to be meaningful. The value of 0.02 S/m was chosen as being greater than the standard deviation of the measurement of conductivity in white matter,^11^ while decreases less than this (non‐responders group) are within the standard deviation and may therefore only represent measurement variability.

Linear regression analyses of differential conductivity maps and their corresponding mean breath alcohol content measurements were conducted within each subgroup. Thresholded T maps with T values >2 (*P* < 0.05) were generated for each subgroup, and, additionally, thresholded T maps with T values >3.5 (*P* < 0.001) were generated for the group of strong responders. The minimum cluster size for the conductivity images, without spatial smoothing, was 48 mm^3^. The combination of reduced intensity (*P*‐value) and cluster thresholds has been shown to provide a balance between Type 1 and Type 2 error.^16^

Finally, given the biphasic response to alcohol that has been reported with different responses to rising or falling alcohol levels,^17^, ^18^, ^19^ we examined the group of strong responders to see if there were differences between those with falling concentrations during the scan (N = 20) and those with rising concentrations (N = 12). The breath alcohol content measurements prior to and post the second MRI session were used to distinguish falling and rising concentrations in the strong responders. One participant whose levels were unchanged was excluded from that analysis. Analysis of variance (ANOVA) was applied to the differential conductivity maps of the two subgroups to see if there was a significant difference in conductivity changes between the two subgroups. Thresholded T maps with T values >3.5 were generated (by considering a threshold for statistical significance of *P* < 0.001).

## Results

### Breath Alcohol Levels

Mean breath alcohol levels immediately preceding the scan were 0.062 ± 0.016 g/210 L (median 0.061, range 0.023–0.110), while immediately following the scan, the mean value was 0.050 ± 0.017 g/210 L (median 0.054, range 0.011–0.078). There was some heterogeneity in the response with 34 participants decreasing BrAC over the duration of the scan (range −0.001 to −0.055 g/210 L), 17 increasing BrAC (range 0.002 to 0.018 g/210/L), and one showing no change. Not all participants finished the entire drink. Mean time of food consumption was 2 hours 31 minutes ± 1 hour 40 minutes prior to the sober scan. Exploratory PCA of age, sex, ethnicity, AUDIT score and BrAC before and after the scan generated no principal components, indicating that there was no relationship between these variables. Similarly, membership of tissue conductivity subgroup showed no relationship with any of BrAC, AUDIT score, sex, ethnicity or age.

### Tissue Electrical Conductivity Response to Ethanol Consumption

**FIGURE 2:**
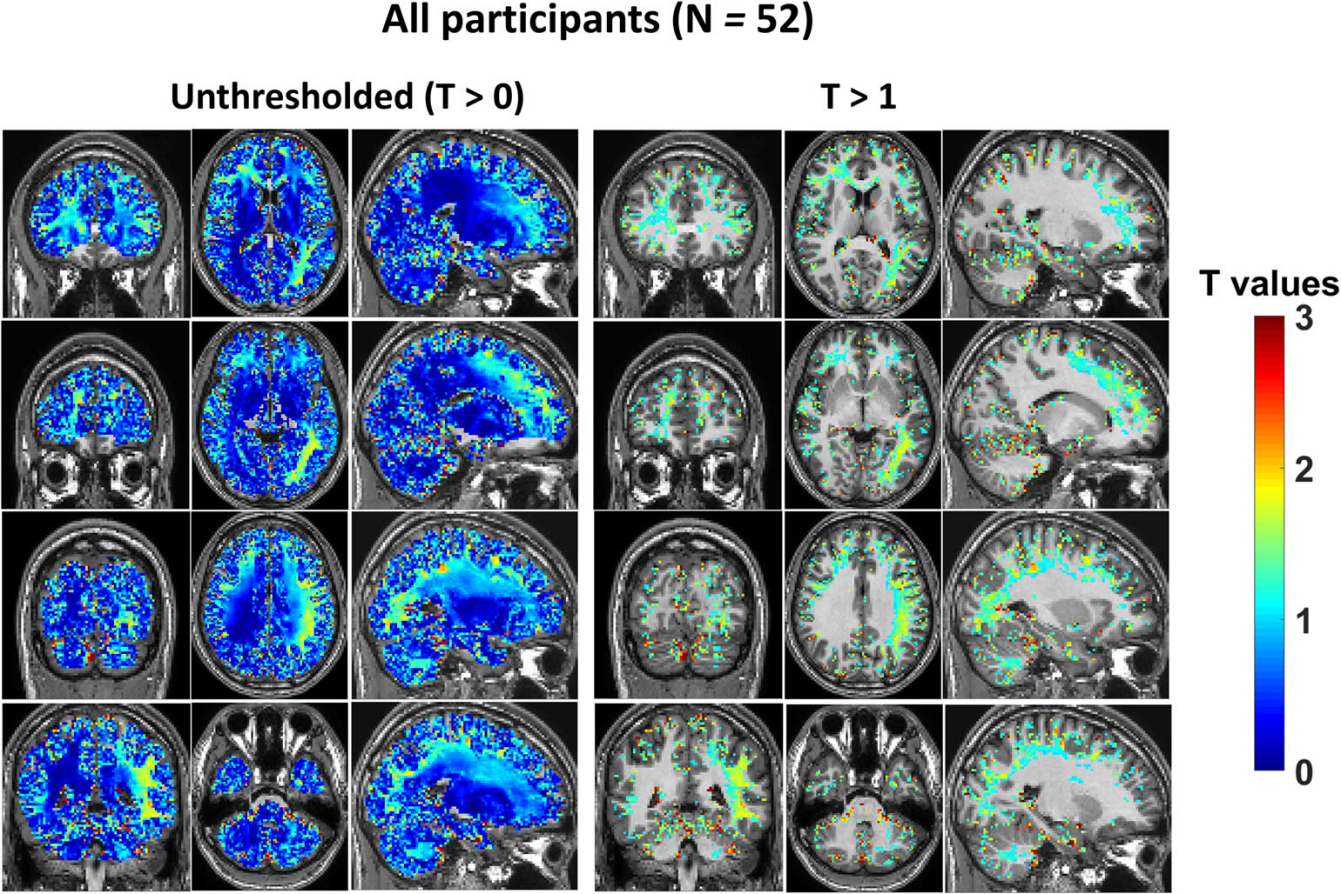
T maps of linear regression analyses of differential conductivity maps and their corresponding mean breath alcohol content measurements from all participants. Maps, normalized in MNI space, on the left are not subject to a threshold and are unsmoothed and show coronal, axial and sagittal views while those on the right are thresholded at T > 1.

When all 52 participants were considered, regression analysis showed areas of decreased tissue conductivity (Fig. 2), none of which were statistically significant. No participant showed significantly increased tissue conductivity following consumption of alcohol.

**FIGURE 3:**
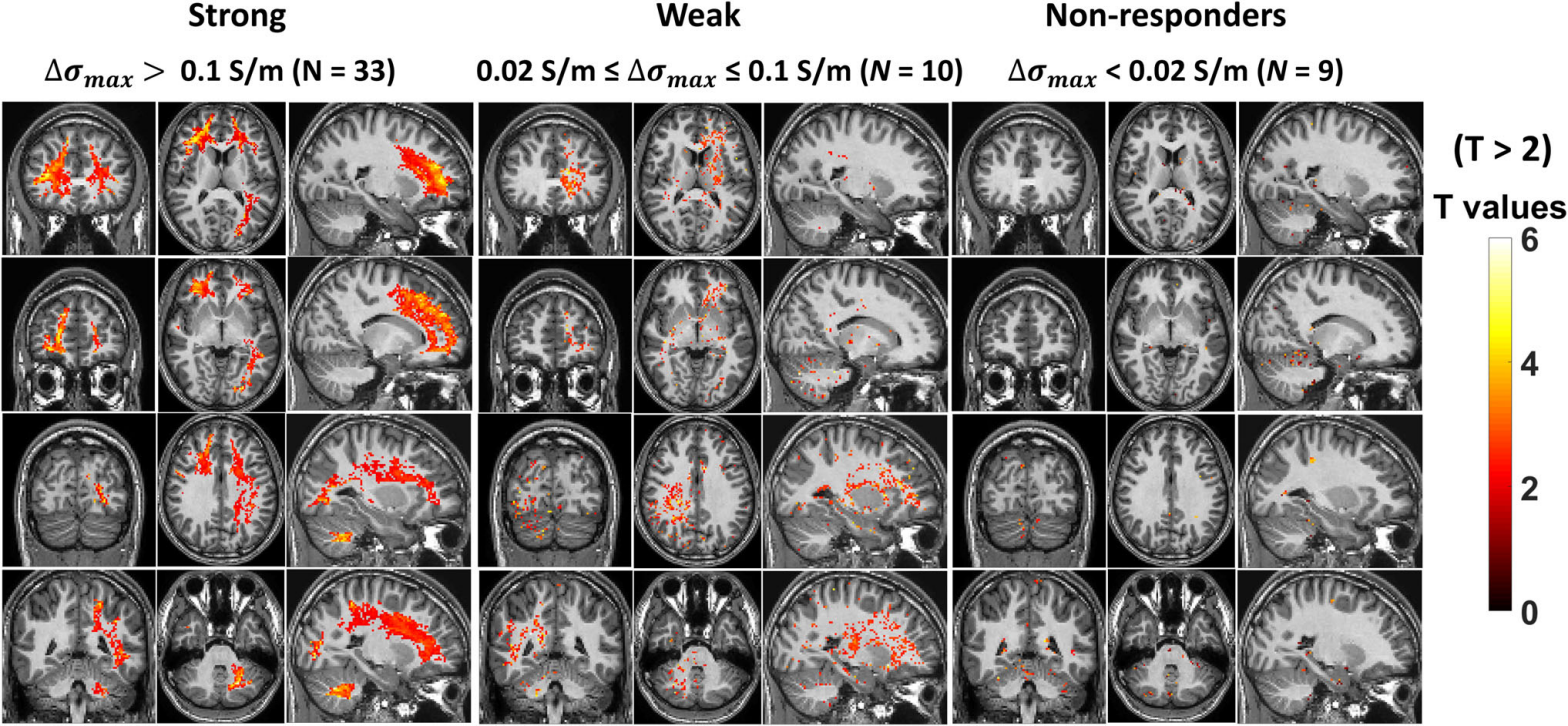
T maps showing significant regional effects of alcohol on tissue conductivity in subgroups of strong, weak and non‐responders to alcohol. Differential conductivity maps from each of strong, weak and non‐responders, and their corresponding breath alcohol measurements were subjected to linear regression analysis. Maps are thresholded at T > 2 (*P* < 0.05). Maps show coronal, axial and sagittal views in MNI space.

**FIGURE 4:**
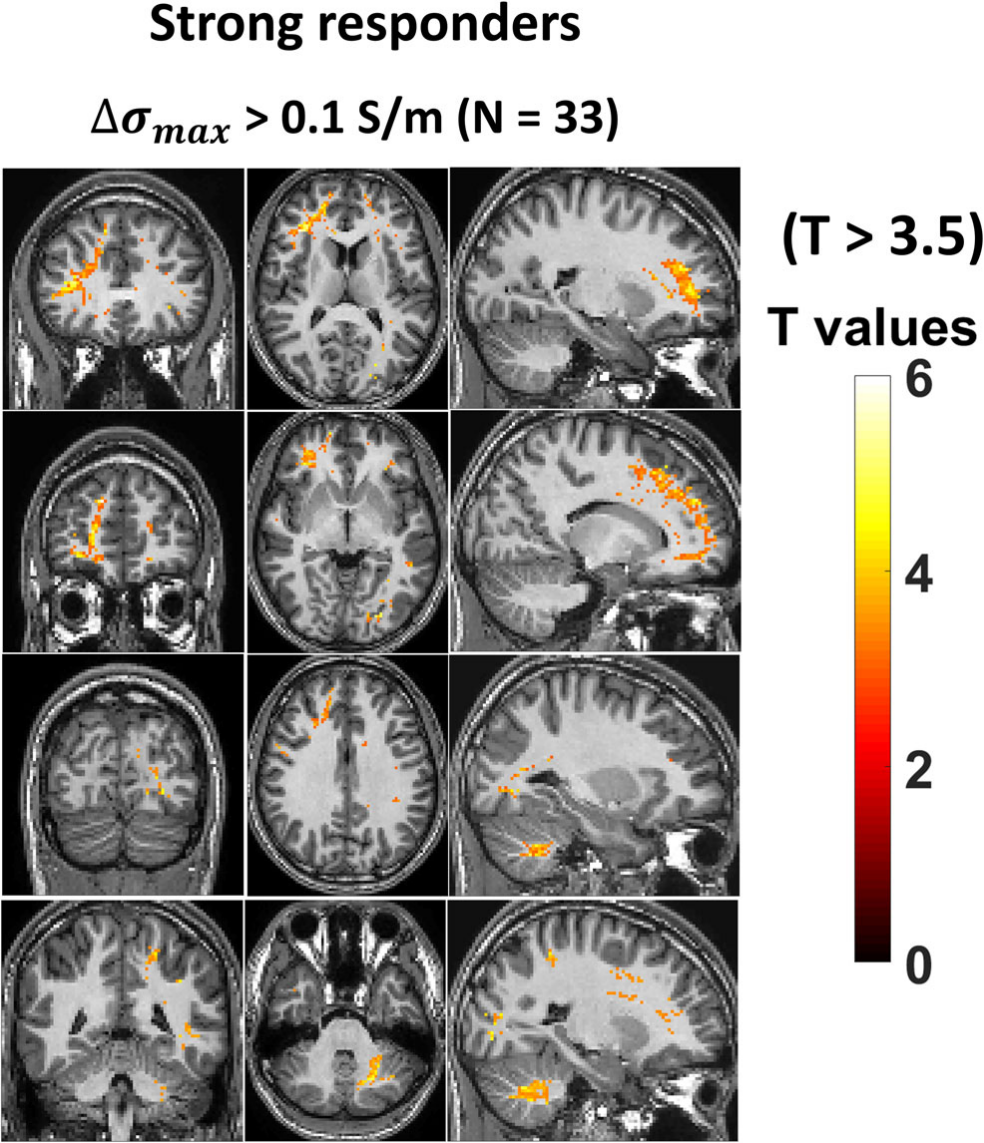
Conductivity changes in strong responders with T ≥ 3.5 (i.e., *P* ≤ 0.001). Differential conductivity maps from the group (N = 33) of strong responders $\left(∆\sigma_{\max}>0.1\;S/m\right)$ and their corresponding breath alcohol measurements were subjected to linear regression analysis and thresholded at T ≥ 3.5. Maps show coronal, axial, and sagittal views in MNI space.

The spatial pattern of decreased tissue conductivity seen in the groups where participants were stratified according to tissue conductivity is shown in Fig. 3. The group (N = 33) with strong decreases in tissue conductivity ($∆\sigma_{\max}>0.1\;S/m$) showed large common areas of significantly decreased (T > 2) tissue conductivity in, for example, the frontal white matter, corpus callosum, cingulum, occipital lobe, and cerebellum. Due to the strict processing approach used (clusterwise thresholding), smaller areas of change in the gray matter were not considered to be statistically significant. The changes in this group can also be visualized with a stricter statistical threshold (T > 3.5) in Fig. 4.

A similar spatial pattern of decrease (albeit weaker) can be seen in those participants with decreased conductivity $\left(0.02\leq ∆\sigma_{\max}\leq 0.1\;S/m\right)$ in the second panel in Fig. 3, while the response shown by those who did not respond ($∆\sigma_{\max}<0.02\;S/m$) is mostly limited to small areas in the cerebellum at the T > 2 threshold (Fig. 3).

There were no significant relationships between group membership (strong, weak, or non‐responder) and gender, Body Mass Index (BMI), BrAC, ethnicity, age, or AUDIT scores.

### Effect of Rising or Falling Alcohol Levels

**FIGURE 5:**
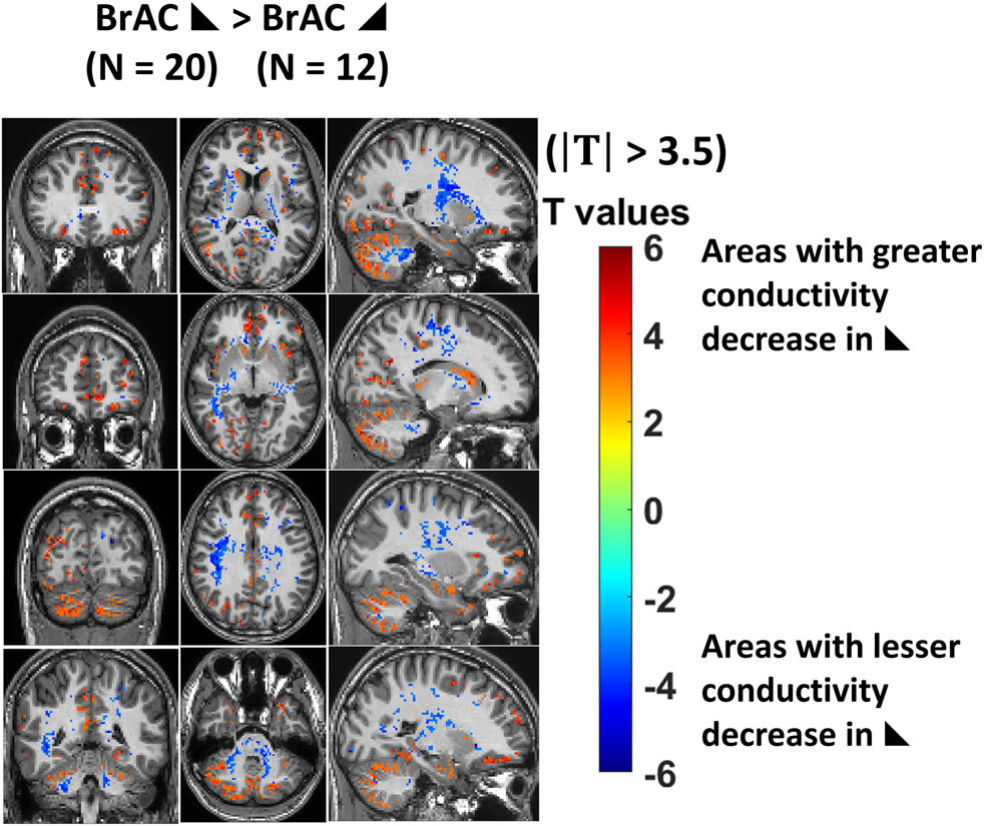
Conductivity maps showing significant differences in strong responders depending on whether breath alcohol levels were rising or falling. Differential conductivity maps from the group of strong responders $\left(∆\sigma_{\max}>0.1\;S/m\right)$ where alcohol levels were rising (N = 12) or falling (N = 20) and their corresponding breath alcohol measurements were subjected to linear regression analysis and thresholded at |T| ≥ 3.5. Areas in red show regions where the decrease in tissue conductivity was greater in those with falling alcohol levels and blue where it was lesser (ANOVA).

The thresholded T‐maps showing different conductivity changes between participants who were strong responders with falling alcohol levels (i.e., with lower BrAC after the scanner session than before the scanner session) vs. those with rising alcohol levels (i.e., with higher BrAC levels after the scan than before the scan) can be seen in Fig. 5. Areas of significantly larger changes in conductivity in those with falling BrAC can be seen in the cerebellar gray matter, while areas of smaller changes compared to those with rising BrAC can be seen in cerebellar and cortical white matter.

## Discussion

This study investigated the impact of ethanol administration on the electrical conductivity of the brain. The majority of participants (42/52) showed significantly decreased conductivity indicating that magnetic resonance electrical properties tomography is sufficiently sensitive to detect and map brain regional effects of alcohol. Different patterns of brain conductivity decrease were generated by whether BrAC was rising or falling, potentially identifying brain substrates of these effects. These results suggest that tissue conductivity may act as a biomarker of brain activity levels and could provide utility in studies of alcohol and other drugs.

Exploratory PCA showed no significant relationship between group membership and BrAC, declared ethnicity, AUDIT score, BMI, or sex. Observations from PET‐FDG uptake studies have reported considerable heterogeneity in the response to ethanol particularly at low concentrations.^7^, ^20^ Heterogeneity in the dopaminergic response to different doses of alcohol has also been reported,^21^ possibly due to the presence of different dopaminergic receptor alleles.^22^ Variants in the GABA(A) α2 gene (GABRA2) have been shown to be associated with individual differences in the response to alcohol,^23^, ^24^, ^25^ as well as impacting the expression level of these receptors.^26^ A previous relationship has been reported between FDG uptake and reported (from autopsy) density of benzodiazepine receptors (α1, α2, α3, or α5‐containing GABA(A) receptors),^27^ yet the cause(s) of decreased conductivity remain to be determined. Certainly ethanol, a known sedative, is known to greatly decrease metabolic activity on acute administration,^1^ and it likely causes this through interactions with receptors.

The spatial pattern of decreased conductivity is very similar to that reported in PET studies using FDG uptake, with decreases in the frontal cortex and cerebellum being the most commonly reported findings.^28^ An MRI diffusion study has reported decreased apparent diffusion coefficient (ADC) in the frontal lobe and cerebellar peduncle after consumption of low‐dose alcohol (0.45 g/kg; Chinese wine along with peanuts) while, additionally, a significantly lower ADC was reported in the thalamus at a higher dose (0.65 g/kg).^29^ Taken together, these results are consistent with the decreases in tissue electrical conductivity reported here, reflecting, respectively, brain metabolic activity and the availability of free water.

We also noted differences in the degree of hemispheric response between the strong and weak responders, with strong responders having a larger conductivity decrease in the left hemisphere, particularly dorsally, while weak responders showed decreases more on the right side dorsally. Differential responses to ethanol in the occipital area have been reported previously, with the fMRI blood oxygen level dependent (BOLD) response to primary visual stimulation on fMRI showing a stronger reduction of the response in the right hemisphere^30^ and another study showing a reduction in the right hemisphere response to the Go/No‐Go task using near‐infrared spectroscopy (NIRS).^31^ It has previously been reported that different blood flow responses have been seen in those with a low response to alcohol vs. those with a high response in response to cognitive tasks under conditions where no alcohol is involved.^32^ This suggests that the pattern of decreased conductivity in strong vs. weak responders may reflect intrinsic differences between these two groups.

The lack of gray matter changes could be attributed to the processing method (clusterwise thresholding) used here. The smaller areas of decreased conductivity in gray matter were most likely removed by this approach. Another reason for the lack of gray matter changes could be due to the reported biphasic response to alcohol,^6^, ^33^ with changes in gray matter becoming apparent in the strong responders group when the direction (increase vs. decrease) of BrAC during the scan was considered.

Differences in physiological, behavioral, and cognitive performance have been reported depending on whether alcohol levels were rising or falling,^17^, ^18^, ^19^, ^34^, ^35^ which suggests that different areas of the brain are affected by the direction of alcohol concentration changes. The gray matter in the cerebellum was particularly impacted by falling alcohol levels, with a greater decrease in conductivity under falling conditions among the strong responders. Cerebellar motor and cognitive functions are reported to recover to drug‐free levels under falling conditions, but errors caused by alcohol fail to recover.^36^ This is in line with the role of the cerebellum as a predictor–corrector,^37^ and it may highlight the role of the time course of alcohol intoxication in understanding human response to alcohol.

No participant showed any increase in electrical conductivity in this study, suggesting that the decrease seen across most participants is probably not a random outcome. Test–retest reliability of tissue conductivity measures has been found to be high.^11^ Tissue conductivity has been shown to be sensitive to increased brain activity, with increases in conductivity reported in activated areas of the brain.^9^ This raises the intriguing possibility that tissue conductivity measures may be a sensitive measure of brain activity and could, for example, substitute for FDG uptake studies, or show utility in studies of pharmacological activity/neurological impairment. The measurement conducted here was short (4 minutes), non‐invasive, and is repeatable multiple times, unlike PET scanning, and provides data at 1 mm isotropic spatial resolution.

### Limitations

While measures of conductivity have been shown to be highly repeatable with minimal test–retest changes,^11^ a limitation of this study was the lack of a placebo arm. Response to alcohol is known to be subjective,^38^ and it is also related to expectation of a response that could lead to over‐compensation in a placebo condition.^39^ The study involved only 52 participants. Not all subjects were able to consume the full amount of alcohol and there was consequently some variability in BrAC levels. Although the actual levels did not appear to show any statistically significant relationship with the tissue conductivity outcomes, there were differences between participants as to whether alcohol concentrations were rising or falling during the second scan.

## Conclusion

Ethanol may reduce brain tissue conductivity in a participant‐dependent and spatially dependent fashion, with frontal white matter and cerebellar areas particularly affected.
